# Making scents of mosquito lures: semiochemical attractants for adult mosquitoes

**DOI:** 10.1186/s13071-026-07488-w

**Published:** 2026-07-09

**Authors:** Nyasha K. T. Thomas, Colin Berry, Islam S. Sobhy

**Affiliations:** 1 School of Biosciences, Cardiff University, Cardiff, UK; 2 School of Natural Sciences, University of Chester, Chester, United Kingdom

**Keywords:** Vector control, Trap, Odour, Behaviour, Mating, Oviposition, Foraging

## Abstract

Mosquito-borne diseases continue to pose a global health threat, particularly in tropical and subtropical regions. As increasing insecticide resistance and ecological concerns challenge traditional vector control methods, semiochemical attractants have emerged as a promising enhancement for surveillance and population management. This review compiles recent advances in the chemical ecology of adult mosquitoes, focusing on volatile organic compounds (VOCs) that influence key behaviours: sugar foraging, blood-feeding, mating and oviposition. We assess the behavioural relevance, chemical composition and experimental validation of attractant blends across major mosquito vector genera, including *Aedes*, *Anopheles* and *Culex*. Special attention is given to host- and microbe-derived VOCs, pathogen-modulated odours, plant-based lures and conspecific cues. By comparing the efficacy of synthetic and natural attractants and highlighting methodological limitations in VOC collection and testing, we identify knowledge gaps and propose directions for developing species- and context-specific lures. This comprehensive review provides a foundation for integrating attractant-based tools into sustainable vector control strategies.

**Graphical Abstract:**

**Figure:**
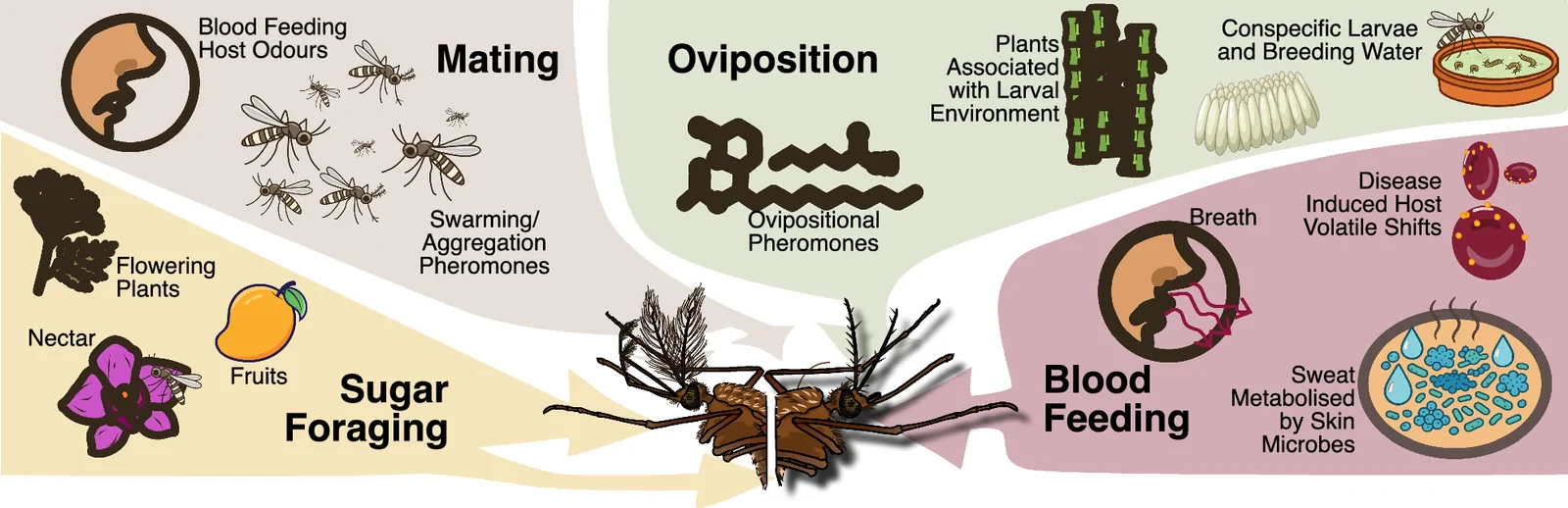

## Background

Mosquitoes are major disease vectors, transmitting infections such as malaria, Zika and dengue through blood-feeding. The genera *Aedes*, *Culex* and *Anopheles* are particularly known for spreading these diseases, primarily affecting low- and middle-income countries in tropical and subtropical regions [1–3]. Mosquito-borne diseases are estimated to infect up to 700 million people annually, resulting in over 1 million deaths and billions of dollars in economic costs [2, 4–6]. Rising urbanisation, global travel and climate change are expanding mosquito habitats and increasing disease transmission [7–9].

Disease control efforts currently focus on monitoring vector populations and reducing human–mosquito interactions through insecticidal agents distributed by indoor spraying and treated nets [10]. Growing behavioural and biological resistance to insecticides in vector populations means that these methods are becoming less effective [11]. These strategies carry significant economic, logistical and environmental burdens, including high implementation costs, challenges in deploying them effectively in the most affected regions and harmful effects on non-target organisms and ecosystems through bioaccumulation [5, 12, 13]. Control via medical interventions and personal protection remains ineffective owing to a lack of distribution and improper implementation in affected areas [14, 15]. Alternatively, environmentally friendly bio-sourced insecticides promise to reduce environmental and non-target organism impacts and carry a lower risk of resistance development; however, their widespread adoption remains constrained by difficulties in formulation, delivery and achieving effective contact with target mosquito populations [16].

**Fig. 1:**
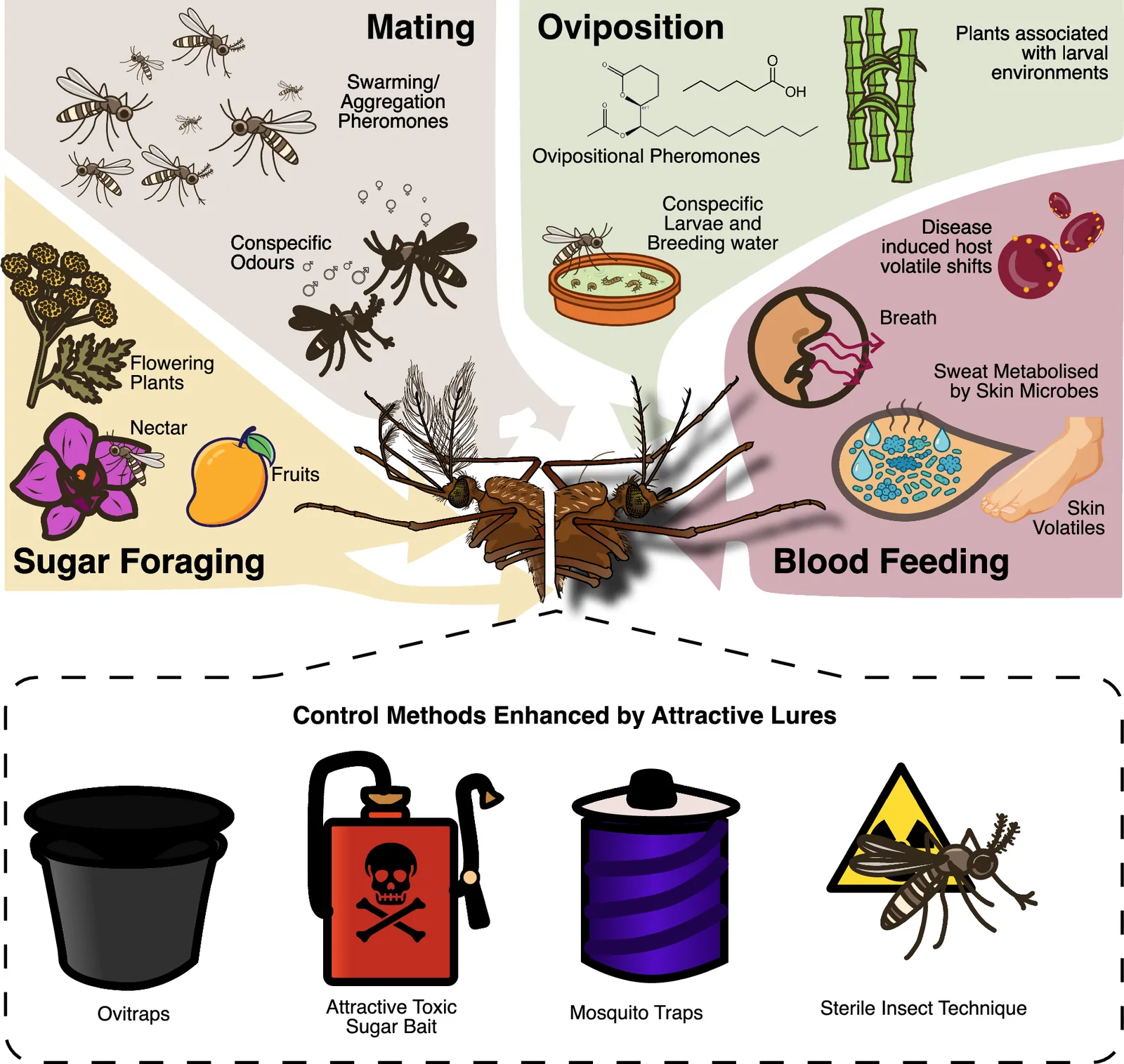
Conceptual overview of semiochemical attractants driving mosquito behaviours and their application in vector control strategies. Schematic representation of the major classes of semiochemical attractants associated with key mosquito behaviours across the life cycle, including blood-feeding, sugar foraging, oviposition and mating. Host-seeking behaviour of female mosquitoes is mediated by skin-derived volatiles, breath cues, microbially modified sweat and pathogen-induced changes in host odours. Sugar foraging is driven by plant-derived volatiles from plant flowers, nectar and fruit, attracting both male and female mosquitoes. Oviposition site selection is influenced by a combination of ecological cues, including conspecific-derived signals, plant-associated compounds characteristic of breeding habitats and volatiles from larval water and associated microbial communities, which modulate the behaviour of gravid female mosquitoes. Mating behaviour is primarily associated with short-range aggregation and swarming pheromones that impact both mosquito sexes. These behaviour-specific attractants can be integrated into control strategies, including odour-baited traps for surveillance and population suppression, ovitraps targeting gravid females, attractive toxic sugar baits (ATSBs) for exploiting sugar-feeding behaviour and sterile insect technique (SIT) programmes that enhance mating efficiency. By linking attractant type to behavioural and physiological states, semiochemical-based approaches provide a framework for targeted, species-specific intervention within integrated vector management

The behaviour and life history traits of mosquitoes are closely tied to their ecological success and impact as disease vectors. In fact, the rise in anthropophilic mosquito behaviour in *Aedes* mosquitoes has been linked to the development of insecticidal resistance [17]. Mosquito life cycle, from aquatic larvae to flying adults, as well as behaviours such as breeding patterns and host preferences, offer opportunities for impactful control interventions. Olfaction is a primary cue used across mosquito life cycles to detect and interpret environmental data, guiding a range of behaviours, including mating, blood-feeding, foraging and oviposition. Semiochemicals play an increasingly important role in mosquito surveillance and control, offering environmentally targeted alternatives or complements to conventional traps and insecticides [18]. By exploiting the chemical cues mosquitoes use to locate hosts, mates, oviposition sites and sugar resources, semiochemical-based tools enable species-, sex- and physiological-state-specific targeting, thereby improving both surveillance sensitivity and control efficiency (Fig. 1) [19, 20]. Chemical lures are particularly advantageous because they offer greater specificity as well as improved chemical consistency and longevity compared with organic substrates [21–24]. For example, host-derived odour lures are effective for targeting females seeking a blood meal, whereas larval habitat-associated odours are better suited for ovitraps targeting gravid females (Fig. 1) [25]. The identification and optimisation of such lures rely on integrated behavioural, chemical and electrophysiological approaches, supporting the development of efficient attractant-based tools that reduce selection pressure for resistance and fit within suitable vector management frameworks.

The databases Web of Science, PubMed and Science Direct were searched for peer-reviewed articles published between 1970 and 2025 using the terms ‘mosquito’, ‘attractants’ and ‘volatiles’. Such a time frame was selected to coincide with the earliest period in which gas chromatography–mass spectrometry (GC–MS) was routinely applied to identify mosquito semiochemicals, ensuring methodological comparability across the included literature, while capturing the full empirical history of the field. Duplicates were removed within Zotero (www.zotero.org). The artificial intelligence (AI) tool ASReview (https://asreview.nl/) was used to screen collected publications on the basis of their title and abstracts using the following inclusion criteria: (1) a focus on blood-feeding mosquitoes as the target; (2) identification of semiochemical compounds and blends comprising attractive substrates; and (3) attraction of mosquitoes to semiochemicals tested. Studies not following the above criteria, not peer-reviewed or not in English were excluded.

This review critically examines the identification of semiochemical attractants in mosquito control, focusing on chemical cues that mediate key adult mosquito behaviours. Despite growing interest in attractant-based interventions, current research remains fragmented, with a limited understanding of the ecological and behavioural specificity of many identified volatiles. This review article addresses that gap by assessing existing studies to evaluate the efficacy and applicability of known attractants across mosquito species and contexts. It offers detailed insights into the behavioural relevance, chemical composition and experimental validation of these lures, providing a comprehensive resource for the development of next-generation vector surveillance and control tools.

## Blood-feeding and host location-associated odours

Female mosquitoes spread disease by feeding on infected hosts and transferring pathogens during subsequent blood meals. Blood-feeding is essential for egg development in many species, making olfactory cues for host location crucial for the production of successive generations [10, 26, 27]. Odours act as the primary signal for host location, not only indicating the presence of a host to a searching female mosquito but also its species and quality. Intra-specific variation in feeding behaviour and host preference complicates the development of universal blood-host attractant formulations while simultaneously enabling species-specific targeting [26, 28]. Pronounced interspecific differences in host selection, from humans to birds to reptiles, drive substantial variation in olfactory responses among blood-feeding mosquitoes. Research primarily focuses on the olfaction and control of anthropophilic vectors such as *Aedes aegypti* (Linnaeus, 1762), *Aedes albopictus* (Skuse, 1894), *Anopheles gambiae* Giles, 1902, *Anopheles funestus* Giles, 1900, and *Culex pipiens* Linnaeus, 1758 (Diptera: Culicidae).

Carbon dioxide (CO_2_) is a key long-range cue for all blood-feeding mosquitoes [29, 30], though its use in traps is impractical in many settings. Short-range cues such as body heat, moisture and volatile organic compounds (VOCs) emanating from skin and breath further guide mosquitoes [31]. Host skin and breath VOCs are an important source of potential mosquito attractants. Human odours, particularly from feet and sweat, contain key attractants such as l-(+)-lactic acid, ammonia and octenol, which are especially attractive when co-presented with CO_2_ [32–35]. These odours are largely shaped by skin-associated microbial metabolism, which generates a distinct volatile profile that enables mosquitoes to differentiate between hosts [36–38]. Key microbially derived kairomones, such as ammonia, increase the attractiveness of human volatiles and can even render otherwise unattractive blends attractive [34, 39].

The BG-lure (Biogents, Thailand), a synthetic human-odour mimic comprising ammonium hydrogen carbonate (NH_4_HCO_3_), hexanoic acid and l-(+)-lactic acid, is widely applied in surveillance but remains less competitive than natural human odour, likely owing to its reduced chemical complexity [40, 41]. Although initially developed as a lure for *Aedes* mosquitoes, the BG-lure also attracts *Culex* mosquitoes effectively across a wide range of concentrations [42]. The Mbita Lure (BG-MB5) (Biogents, Thailand), which targets *Anopheles* spp. similarly, contains not only ammonia and l-(+)-lactic acid but also tetradecanoic acid and 3-methyl-butanol [43, 44].

Differences in the attractiveness of individual hosts stem from variations in the composition and abundance of these chemical signals [32, 45–48]. Compounds including l-(+)-lactic acid, 2-phenylethanol, 2-methybutanoic acid and tetradecanoic acid are more abundant in the volatile profiles of individuals more attractive to *An. gambiae*, while limonene, 2-phenylethanol and 2-ethyl-1-hexanol were more abundant in those less attractive [49]. Compounds including dodecanol and 3-methylbutanol are relatively overproduced by human skin microbiota associated with individuals highly attractive to *Aedes* females; however, the behavioural effects on *Aedes* of varying the abundance of specific compounds within these complex odour blends have not yet been fully elucidated [50]. Blood-seeking females have evolved to seek out specific compounds and chemical blends that signal a suitable blood host, specific metabolic activity and those compounds that are reliably associated with host availability [51]. Choosing the correct blood host, even between individuals of the same species, is paramount for the blood-feeding female, as variations in haemoglobin, plasma proteins and immune factors impact their reproductive output. Modulation of these influential compounds can alter the attractiveness of human hosts: The addition of compounds more abundant in less attractive individuals reduces the attractiveness of more attractive humans and vice versa [52, 53].

Increasing the chemical complexity of host-based lures through the addition of other host-derived compounds, including fatty acids (e.g. 3-methyl-butanoic acid, propionic acid and octanoic acid), and alcohols (e.g. 3-methyl-1-butanol) further enhances blend attractiveness and is a crucial consideration in making more competitive lures (Table 1A) [35, 44, 54–58]. Bacterial isolates present on skin, such as *Brevibacterium linens* (Wolff), a bacterium responsible for human foot odour and present in Limburger cheese, has shown some proficiency as a lure. Fatty acids produced by this bacterial species have been shown to lure *An. gambiae*, *C. pipiens* and *Ae. aegypti* in the field [59–61]; however, they struggle to compete with human host odours, reinforcing the contribution of the whole skin microbiota (Table 1A), [33, 34, 62].

**Table 1:** Chemical compounds and blends tested as mosquito attractants. Compounds and blends mimic odours associated with blood-feeding (A), mating (B), oviposition (C), sugar foraging (D) and other origins (E). The odour source, experimental study, mosquito species and sex, compounds tested as well as compounds or blends identified as attractive (or repellent where indicated) are listed.

Odour source	Testing environment	Tested compounds	Tested species	Tested mosquito sex	Attractive compounds/blends	Reference
A. Blood feeding						
Rat volatiles	In vitro	Phenol, 4-methylphenol, 4-ethylphenol, and indole	*Aedes albopictus*	F	4-Methylphenol + 4-ethylphenol/4-methylphenol + 4-ethylphenol + indole/4-methylphenol + 4-ethylphenol + indole + phenol	[178]
Human host volatiles + carboxylic acids from Limburger cheese	In vitro	Ammonia, lactic acid, 12 carboxylic acids (ethanoic acid, propanoic acid, 2-methylpropanoic acid, butanoic acid, 3-methylbutanoic acid, pentanoic acid, hexanoic acid, octanoic acid, decanoic acid, dodecanoic acid, tetradecanoic acid, hexadecenoic acid)	*Anopheles gambiae*	F	Ammonia/ammonia + 12 carboxylic acids/ammonia + lactic acid + CO_2_/ammonia + lactic acid + 12 carboxylic acids	[34]
	In vivo	l-lactic acid, CO_2_, propanoic acid, hexanoic acid, valeric acid, butanoic acid	*Anopheles gambiae* *Anopheles funestus*	F	l-lactic acid + CO_2_	[33]
Human skin/sweat volatiles	Semi-field In vivo	CO_2_, ammonia, l-lactic acid, propionic acid, butanoic acid, pentanoic acid, 3-methylbutanoic acid, heptanoic acid, octanoic acid, tetradecanoic acid	*Anopheles gambiae*	Mixed	CO_2_ + ammonia + l-lactic acid + propionic acid + butanoic acid + pentanoic acid + heptanoic acid + octanoic acid + tetradecanoic acid ± 3-mthylbutanoic acid	[55]
	In vitro + in vivo	(*E*)-3-methyl-2hexenoic acid, (*Z*)-3-methyl-2hexenoic acid, 7-octenoic acid, CO_2_	*Anopheles gambiae*	F	7-Octanoic acid + CO_2_	[179]
	In vitro	Benzaldehyde, sulcatone, octanal, nonanal, linalool, menthol, naphthalene, decanal, geranyl acetone	*Aedes aegypti*	F	Repellent: sulcatone/octanal/nonanal/decanal/geranyl acetone	[52]
	In vitro	Ammonia, lactic acid, 12 carboxylic acids	*Anopheles gambiae*	F	Ammonia/12 carboxylic acids/ammonia + lactic acid + 12 carboxylic acids	[83]
	Semi-field	Standard synthetic blend (SB): ammonia, (*S*)-lactic acid, tetradecanoic acid, CO_2_ Isovaleric acid, 4,5 dimethylthiazole, 2-methyl-1-butanol, and 3-methyl-1-butanol	*Anopheles gambiae*	F	SB + 3-methyl-1-butanol	[56]
	In vitro	Ammonia, CO_2_, lactic acid, octanoic acid, 1-octene-3-ol, 1-nonanol, 1-hexanol, 1-hexanoic acid, 1-hexene-3-ol, 1-heptaldehyde, hexenal, γ-terpinene, ß-pinene, α-pinene, myristic acid, 3-methyl indole, indole, 3-undecanone, *p*-cymol, limonene, 5-isopropyl-2-methyl phenol, inalool, 1,4-cineole, Menthone	*Aedes aegypti*	F	1-Octene-3-ol/lactic acid/1-nonanol/ ammonia/myristic acid/CO_2_/myristic acid + lactic acid + 1-octen-3-ol + CO_2_/myristic acid + ammonia + CO_2_/myristic acid + 1-octen-3-ol + CO_2_/myristic acid + lactic acid + ammonia + 1-octen-3-ol + CO_2_/myristic acid + lactic acid + CO_2_	[35]
	In vitro	1-octen-3-ol, caffeic acid, nonanoic acid, heptanoic acid, propionic acid, acetic acid, formic acid, butyric acid, ethyl acetate, ethyl alcohol, sulcatone	*Aedes aegypti*	F	Sulcatone/formic acid/acetic acid/ethyl alcohol/butyric acid/nonanoic acid/caffeic acid/1-octen-3-ol/heptanoic acid/acetic acid + ethyl acetate + ethyl alcohol + sulcatone/acetic acid + ethyl acetate + ethyl alcohol/acetic acid + formic acid/nonanoic acid + acetic acid + formic acid + ethyl acetate + ethyl alcohol/acetic acid + ethyl alcohol/acetic acid + ethyl acetate/acetic acid + formic acid + ethyl acetate + ethyl alcohol	[86]
	Semi-field + in vivo	Mbita base blend (MB): ammonia + l-lactic acid + tetradecanoic acid + 3-methyl1-butanol + CO_2_ butyl-2-methylbutanoate, 2-pentadecanone, 1-doddecanal, butan-1-amine	*Anopheles gambiae* *Anopheles funestus* *Culex* spp.	F	MB + 2-pentadecanone/MB + 1-doddecanal/MB + butan-1-amine	[44]
	In vitro	Propionic acid, benzaldehyde, 1-octen-3-ol, geranyl acetone, hexanal, myristic acid, sulcatone, heptanoic acid, octanoic acid, *p*-cresol, butyric acid	*Culex pipiens*	F	Propionic acid/benzaldehyde/1-octen-3-ol/geranyl acetone/hexenal/myristic acid/benzaldehyde + 1-octen-3-ol/geranyl acetone + myristic acid + propionic acid/benzaldehyde + 1-octen-3-ol + hexanal + propionic acid/geranyl acetone + benzaldehyde + 1-octen-3-ol + hexanal + myristic acid/benzaldehyde + 1-octen3-ol + hexanal + myristic acid + propionic acid	[87]
	In vitro	Lactic acid, octenol, isovaleric acid, isoamyl alcohol, octenol, myristic acid, ammonium hydroxide	*Aedes aegypti*	F	Lactic acid, octenol, isovaleric acid	[42]
			*Culex quinquefasciatus*		Lactic acid, octenol, isovaleric acid	
			*Anopheles minimus*		Lactic acid, octenol, isovaleric acid	
	In vivo	Hexanal, heptanal, hexanoic acid, octanal, nonanal, decanal and undecanal, propionic acid, 3-methylbutyric acid, and geranyl acetone	*Aedes aegypti*		Nonanal + octanal + CO_2_/hexanoic acid + CO_2_	[41]
		Butyric acid, i-valeric acid, octanal, nonanal, decanal, dodecanal, undecanal, tridecanal, 4-ethoxyacetophenone, 4-ethylacetophenone, 2-methylphenol	*Anopheles gambiae*		Butyric acid, i-valeric acid, octanal, nonanal, decanal, dodecanal, undecanal, tridecanal	[46]
Human skin microbiota	In vivo	1-Methyl-1-butanol + CO_2_	*Anopheles gambiae*	F	Not attractive	[180]
	In vivo	CO_2_, 1-octen-3-ol, acetone, butyric acid	*Culex quinquefasciatus*	Mixed	CO_2_	[181]
	In vitro	1-Butanol + 2,3-butanedione + 2-methyl-1-butanol + 2-methylbutanal + 2-methylbutanoic acid + 3-hydroxy-2-butanone + 3-methyl-1-butanol + 3-methylbutanal + 3-methylbutanoic acid + benzene ethanol	*Anopheles gambiae*	F	1-butanol + 2,3-butanedione + 2-methyl-1-butanol + 2-methylbutanal + 2-methylbutanoic acid + 3-hydroxy-2-butanone + 3-methyl-1-butanol + 3-methylbutanal + 3-methylbutanoic acid + benzene ethanol	[36]
	In vitro	Standard synthetic blend (SB): ammonia, (l)-(+)-lactic acid, tetradecanoic acid Butyl 2-methylbutanone, butyl butyrate, butyl acetate, 2-pentadecanone, butyl isobutyrate, dimethyldisulfide	*Anopheles gambiae*	F	SB + butyl 2-methylbutanoate SB + butyl butyrate SB + butyl acetate SB + dimethyldisulfide SB + butyl isobutyrate	[81]
	In vitro Semi-field	Standard synthetic blend (SB): ammonia, (l)-(+)-lactic acid, tetradecanoic acid + CO_2_ 1-butanol, 2-methylbutanoic acid, 3-hydroxy-2-butanone, 3-methyl-1-butanol, 3-methylbutanal, 2,3-butanedione, 3-methylbutanoic acid, 2,3-butanedione, 3-methylbutanol and 2-phenylethanol	*Anopheles gambiae*	F	SB + 3-methyl-1-butanol/1-butanol/2-methylbutanoic acid SB + 3-hydroxy-2-butanone SB + 3-methylbutanal SB + 2,3-butanedione/3-methyl-1-butanol/3-methylbutanoic acid	[54]
	In vitro	Standard synthetic blend (SB): ammonia, (*S*)-lactic acid, tetradecanoic acid + CO_2_ 3-methylbutanoic acid, 3-methyl-1-butanol, 3-methylbutanal, butan-1-amine	*Anopheles coluzzii*	F	SB + 3-methyl-1-butanol/3-methylbutanol/butan-1-amine SB + CO_2_ + 3-methyl-1-butanol + butan-1-amine	[57]
Plasmodium-infected mice volatiles	In vitro	Hexanoic acid, 2-phenylethanol, 2-methyl butanoic acid, 3-methyl butanoic acid, tridecane, benzothiazole	*Anopheles stephensi*	F	When added to ‘healthy’ host odour profiles: hexanoic acid, 2-methyl butanoic acid, 3-methyl butanoic acid, tridecane	[63]
Plasmodium-infected human volatiles	In vitro	MB5 synthetic base (MB5): ammonia comprising ammonia, l-(+)-lactic acid, tetradecanoic acid, 3-methyl-1-butanol, and butan-1-amine (*E*)-2-octenal/(*E*)-2-decenal/heptanal/octanal/nonanal	*Anopheles gambiae*	F	MB5 + heptanal/MB5 + heptanal octanal + nonanal + (*E*)-2-octenal, + (*E*)-2-decenal	[75]
	In vivo	(±)-α-Pinene, (−)-β-pinene, (±)-limonene, octanal, nonanal, decanal	*Anopheles gambiae*, *Anopheles arabiensis*, *Anopheles pharoensis*		(±)-α-Pinene, (−)-β-pinene, (±)-limonene, octanal, nonanal, decanal	[78]
Zika flavirus-infected human volatiles	In vitro	Acetophenone, decanal, styrene	*Aedes aegypti*	F	Acetophenone	[77]
Skin volatiles of sheep, cows, donkeys, goats and humans	In vitro In vitro	Heptanal, octanal, nonanal, decanal	*Aedes mcintoshi* *Ae. ochraceus*	F	Heptanal + octanal + nonanal + decanal + CO_2_	[58]
Volatiles from humans, cattle and chickens	In vitro	(*R*)-1-octen-3-ol Human (0.06 ± 0.04 ng min −1) Cattle (0.6 ± 0.35 ng min − 1) Chicken	*Aedes aegypti* *Anopheles coluzzii* *Culex quinquefasciatus*	F	(*R*)-1-octen-3-ol + CO_2_	[182]
Human and avian volatiles	In vitro	Sulcatone, nonanal, decanal, geranylacetone, heptanal, octanal	*Culex quinquefasciatus*	F	Nonanal + CO_2_	[70]
Avian volatiles	In vitro	Hexananl, nonanal, α-pinene, nenzaldehyde, β-myrcene, meso-2,3-butanediol, 2,3-butanediol, 2,3-docosanediol	*Culex quinquefasciatus* *Culex tarsalis Coquillett* *Culex nigripalpus* *Aedes aegypti*	F	Not attractive	[65]
Avian and bovine volatiles	In vitro	Acetic acid, butanoic acid, heptanoic acid, lactic acid, 3-methyl butanoic acid, myristic acid, palmitic acid, stearic acid, salicylic acid, propionic acid, benzoic acid, carbon disulfide, dimethyl disulfide, dimethyl trisulfide, ethyl disulfide, methyl propyl disulfide, methyl sulfide	*Aedes aegypti*	F	Acetic acid/lactic acid/carbon disulfide/dimethyl disulfide/methyl sulfide/benzoic acid/butanoic acid/heptanoic acid 3-Methylbutanoic acid/myristic acid/palmitic acid/salicylic acid/stearic acid/methyl propyl disulfide	[183]
			*Culex quinquefasciatus*		Bovine blood/lactic acid/acetic acid/palmitic acid/stearic acid Dimethyl trisulfide/methyl propyl disulfide	
			*Culex nigripalpus*		Myrtistic acid/dimethyl sulfide/methyl propyl disulfide/lactic acid/stearic acid/dimethyl trisulfide/ethyl disulfide/methyl propyl disulfide	
Candidate behaviourally disruptive organic compounds	In vitro and semi-field	Standard synthetic blend (SB): ammonia + lactic acid + tetradecanoic acid + CO_2_ 2-Methylpropanal, octanal, nonanal, decanal, 4-methylcyclohexanol, 3-heptanol, 3-octanol, 1-butylamine, 1-pentylamine, acetophenone, benzaldehyde, eugenol, 2-phenylethanol, 2,ethyltoluene, 2-oxopentanoic acid, butylpropionate, isoamylpropionate, heptylformate, methyl-trans-3-hexenoate, 2-acetylthiophene, 4,5-dimethylthiazole, pyridine, 2-octanone, 2-nonane, δ-undecalactone, δ-decalactone, linalool oxide	*Anopheles gambiae*	F	Attractive: SB + 3-heptanol/SB + 2-methylpropanal/SB + 4,5-dimethylthiazole	[184]
(B) Mating						
Adult conspecifics	In vitro	2,6,6-Trimethylcyclohex-2-ene-1,4-dione (ketoisophorone)/2,2,6-trimethylcyclohexane-1,4-dione (ketoisophorone analogue)/1-(4-ethylphenyl) ethanone	*Aedes aegypti*	Mixed	Attractive to F: ketoisophorone/1-(4-ethylphenyl) ethanone Attractive to M: ketoisophorone	[96]
Swarming adult male conspecifics	In vitro and vivo	3-Hydroxy-2-buranone/sulcatone/octanal/nonanal/decanal	*Anopheles arabiensis* *Anopheles gambiae* *Anopheles coluzzii* *Anopheles merus* *Anopheles funestus*	Mixed	3-Hydroxy-2-buranone + sulcatone + octanal + nonanal + decanal	[97]
(C) Oviposition						
Conspecific eggs	In vitro	Erythro-6-acetoxy-5-hexadecanolide	*Culex pipiens*	F	Erythro-6-acetoxy-5-hexadecanolide	[103]
	In vivo	Erythro-6-acetoxy-5-hexadecanolide	*C. quinquefasciatus*	F	Erythro-6-acetoxy-5-hexadecanolide	[104]
		Erythro-6-acetoxy-5-hexadecanolide	*Culex pipiens*	Mixed	Attractive to F: erythro-6-acetoxy-5-hexadecanolide	[185]
	In vitro	6-Hexanolactone, methyl dodecanoate, dodecanoic acid, methyl tetradecanoate, tetradecanoic acid, methyl (*Z*)-9-hexadecenoate, methyl hexadecanoate (*Z*)-9hexadecenoic acid, hexadecanoic acid, methyl (*Z*)-9-octadecenoate, methyl octadecanoate (*Z*)-9octadecenoic acid, octadecanoic acid	*Aedes aegypti*	F	Attractive: dodecanoic acid/(*Z*)-9-hexadecenoic acid Repellent: 6-hexanolactone/methyl dodecanoate/methyl tetradecanoate/ tetradecanoic acid/methyl (*Z*)-9-hexadecenoate/methyl hexadecanoate/hexadecanoic acid/methyl (*Z*)-9-octadecenoate/methyl octadecanoate (*Z*)-9-octadecenoic acid/octadecanoic acid	[105]
Volatiles from conspecific larvae and larval environments	In vitro	(*E*)-2-octenal, decanal, 2,4-dimethylhept-1-ene, 4-cyanocyclohexene, 2,6-dimethyl-7-octen-2-ol, nonanal, 4-(2-methylbutan-2-yl)phenol, camphor, (*E*)-2-nonenal, (*E*)-2-decenal, (*E*)-2-heptenal, unknown branched c12 alkane	*Aedes aegypti*	F	2,4-Dimethylhept-1-ene + 2,6-dimethyl-7-octen-2-ol + camphor + decanal/2,4-dimethylhept-1-ene + (*E*)2-heptanal + nonanal + camphor + (*E*)-2-nonenal + (*E*)-2-decenal + 4-(2-methylbutan-2-yl)phenol/4-cyanocyclohexene + 2,6-dimethyl-7-octen-2-ol + nonanal	[107]
	In vitro	Nonane, 2,4-pentanedione, dimethyl disulfide, dimethyl trisulfide	*Anopheles coluzzii*	F	Attractive: nonane/2,4-pentadione Repellent: dimethyl disulfide/dimethyl trisulfide	[109]
	In vitro	Isovaleric acid/myristoleic acid/tetradecanoic acid/pentadecanoic acids	*Aedes aegypti*	F	Pentadecanoic acid/isovaleric acid + myristoleic acid + tetradecanoic acid + pentadecanoic acids Mix of acids	[108]
Hay infusion volatiles	In vitro and vivo	Dimethyl disulfide/Indole/4-methylphenol/3-methylindole/trimethylamine	*Aedes albopictus*	F	Repellent: 4-methylphenol/3-methylindole	[186]
Rice pollen volatiles	In vitro	ß-caryophyllene, decanal, sulcatone, nonanal, limonene, 3-carene, β-pinene, α-pinene	*Anopheles arabiensis*	F	ß-caryophyllene + decanal + sulcatone + nonanal + limonene + 3-carene + β-pinene + α-pinene	[135]
Maize pollen volatiles	In vitro	benzaldehyde, nonanal, *p*-cymene, limonene and α-pinene	*Anopheles arabiensis*	F	Benzaldehyde + nonanal + *p*-cymene + limonene + α-pinene	[136]
Sugarcane pollen volatiles	In vitro	*o*-Xylene, styrene, (1*R*)-(+)-α-pinene, benzaldehyde, 1,8-cineole, nonanal, undecane, *p*-cymene, *N*-ethylbenzenamine, dibutyl phthalate and eicosane	*Anopheles arabiensis*		Benzaldehyde/nonanal/(1*R*)-(+)-α-pinene/benzaldehyde + nonanal + (1*R*)-(+)-α-pinene, *p*-cymene	[137]
Alfalfa hay volatiles	In vitro	Methyl isovalerate, 1-heptanol, (1*R*)-(+)-α-pinene, phenol, (1*S*)-(−)-linalool, 4-ethylphenol, 4-ethylguaiacol, indole and undecanone	*Culex quinquefasciatus*	F	Methyl isovalerate + 1-heptanol + (1*R*)-(+)-α-pinene + phenol, (1*S*)-(−)-linalool + 4-ethylphenol + 4-ethylguaiacol + indole + undecanone	[139]
Compounds common in [135–137]	In vitro and vivo	α-Pinene, (*R*)-(+)-limonene, *p*-cymene, nonanal, benzaldehyde	*Anopheles arabiensis* *Anopheles pharoensis* *Culex* spp.	Mixed	α-Pinene + (*R*)-(+)-limonene + *p*-cymene + nonanal + benzaldehyde	[138]
Soil volatiles	In vitro and semi-field	Cedrol	*Anopheles gambiae*	F	Cedrol	[110]
	In vivo and vitro	Geosmin	*Aedes aegypti*	F	Geosmin	[142]
Fermenting molasses volatiles	In vitro	Isoamylacetate, styrene, alpha-pinene, 2-ethyl-6-methyl pyrazine, 2,3,5-trimethylpyrazine, ethyl caprylate, caprolactam, decanoic acid, ethyl ester	*Anopheles coluzzi*	F	CO_2_ + isoamylacetate + styrene + α-pinene + 2,3,5-trimethylpyrazine + ethyl caprylate + caprolactam + decanoic acid + ethyl ester	[187]
Liu-pao tea	In vitro	Cedrol, linalool oxide	*Aedes aegypti* *Aedes albopictus*	F	Cedrol + linalool oxide	[140]
Urea	In vivo	Urea	*Culex tritaeniorhynchus*	F	Urea	[148]
Mosquito odorant-binding protein ligands	In vitro	Acetaldehyde	*Culex quinquefasciatus*	F	Acetaldehyde	[149]
(D) Foraging						
*Senecio madagascariensis* flower volatiles	In vitro	Terpenes (±)-α-pinene, β-pinene, sabinene, and phellandrene and 1-alkenes 1-undecene, and 1-nonene	*Aedes aegypti*	Mixed	1-Nonene attractive to M only	[188]
*Lobularia maritima* inflorescence volatiles	In vitro	Acetophenone, 1-octanol, 2-phenylethanol, benzyl cyanide, and benzyl isothiocyanate, 3-butenyl isothiocyanate, phenylacetaldehyde, 1,2-thiiranpentylnitrile, 4-methyl-pentyl isothiocyanate, hexyl isothiocyanate, and 4-vinyl-2-methoxy phenol, 4-Pentenyl isothiocyanate, 1,2-thiiranhexylnitrile	*Aedes aegypti*	Mixed	Acetophenone to M and F 1-Octanol repellent to F	[189]
Aphid infested honeydew volatiles	In vitro	2,3-Butanediol, 2,3-butanedione, 2,5-dimethylpyrazine, 2-ethylhexanol, 2-methyl-2-buten-1-ol, 2-methylbutanal, 2-methylbutanoic acid, 2-phenylethyl alcohol, 3-hydroxybutanone, 3-methyl-1-butanol, 3-methyl-2-butenal, 3-methyl-3-buten-1-ol, 3-methylbutanal, 3-methylbutanoic acid, butanoic acid, limonene, propanone	*Aedes aegypti*	F	2,3-Butanedione + 2,3-butanediol + 3-hydrocybutanone + 3-methyl-1-butanol + 3-methylbutanoic acid + 2-methylbutanoic acid + 2-ethylhexanol + 2-phenylethyl alcohol	[154]
*Parthenium hysterophorus* plant volatiles	In vitro	α-Pinene, camphene, 1-octen-3-ol, β-pinene, *cis*-β-ocimene, bornyl acetate, α-caryophyllene, hexadecanoic acid, α-linolenic acid	*Anopheles gambiae*	Mixed	α-Pinene, camphene, and *cis*-β-ocimene	[168]
	In vitro	Hexenal, β-pinene, d-limonene, (*E*)-β-ocimene, (*E*)-linalool oxide and (*E*)-β-farnesene	*Anopheles gambiae*	F	Hexanal/(*E*)-linalool oxide/β-ocimene/*(E*)-β-farnesene/hexanal + β-pinene + d-limonene, + (*E*)-β-ocimene + (*E*)-linalool oxide + (*E)*-β-farnesene	[155]
*Acelpias syriaca* plant volatiles	In vitro	2-Phenylethanol, benzyl alcohol	*Culex pipiens*	F	Not attractive	[160]
	In vitro	Benzaldehyde, (*E*)β-ocimene, phenylacetaldehyde, benzyl alcohol, nonanal, and (*E*)-2-nonenal	*Culex pipiens*	Mixed	Benzaldehyde + phenylacetaldehyde + (*E*)-2-nonenal	[161]
*Abelia chinensis* plant volatiles	In vitro	Benzaldehyde, α-pinene, phenyl acetaldehyde, benzyl alcohol, β-caryophyllene, and phenylethyl alcohol	*Culex pipiens*	F	α-Pinene + benzaldehyde + benzyl alcohol + phenyl acetaldehyde + phenylethyl alcohol + β-caryophyllene	[165]
*Silene otites* plant volatiles	In vitro	Phenyl acetaldehyde, veratrole, 2-methoxyphenol	*Culex pipiens*	Mixed	Phenyl acetaldehyde/phenyl acetaldehyde + veratrole + 2-methoxyphenol	[164]
	In vitro	(*Z*)-3-hexen-1-ol, hexanol, (*Z*)-3-hecexenyl acetate, benzaldehyde, benzyl alcohol, phenyl acetaldehyde, acetophenone, linalool oxide, linalool, phenylethyl alcohol, lilac aldehydes, methyl salicylate	*Culex pipiens* *Aedes aegypti*	Mixed	Acetophenone/linalool oxide/phenyl acetaldehyde/phenylethyl alcohol/acetophenone + linalool oxide + phenyl acetaldehyde + phenylethyl alcohol	[163]
Common Phytochemicals	In vitro	(*E*)-β-ocimene/α-pinene/β-pinene/d-limonene/linalool/benzaldehyde/phenyl acetaldehyde/benzyl alcohol/methyl salicylate/hexanol/(*Z*)-3hexen-1-ol/(*Z*)-3-hexenyl acetate/Hexanal/(*E*)-2-hexenal/nonanal/pentane/phenylethyl alcohol/acetophenone	*Culex pipiens*	F	(*E*)-β-ocimene/α-pinene/β-pinene/d-limonene/linalool/benzaldehyde/phenyl acetaldehyde/benzyl alcohol/methyl salicylate/hexanol/(*Z*)-3hexen-1-ol/(*Z*)-3-hexenyl acetate/Hexanal/(*E*)-2-hexenal/nonanal/pentane/phenylethyl alcohol/acetophenone/(*E*)-β-ocimene + linalool + benzaldehyde + hexanol + (*Z*)-3-hexen-1-ol + phenylethyl alcohol	[166]
	In vivo	(*E)*-linalool oxide, (*E*)-ß-ocimene, hexanal, ß-pinene, limonene, (*E)*-ß-farnesene, heptanal, octanal, nonanal, decanal	*Anopheles gambiae* *Anopheles arabiensis* *Anopheles funestus*	Mixed	(*E*)-linalool oxide ± CO_2_/(*E*)-linalool oxide + (*E*)-ß-Ocimene/heptanal + octanal + nonanal + decanal ± (*E*)-linalool oxide/(*E*)-linalool oxide + (*E*)-ß-ocimene + hexanal + ß-pinene + limonene + (*E*)-ß-farnesene	[169]
	In vivo	Linalool oxide	*Aedes mcintoshi* *Aedes ochraceus* *Aedes aegypti*	Mixed	Linalool oxide + CO_2_	[172]
	Semi-field	linalool, 1-hexanol, and phenylacetaldehyde	*Aedes aegypti*	Mixed	Phenylacetaldehyde + 1-hexanol + linalool	[171]
	In vitro	Linalool, geraniol, citronellal, eugenol, anisaldehyde, citral	*Aedes albopictus*	F	Attractive: anisaldehyde/eugenol Repellent: citronellal/linalool/citral/geraniol	[170]
Ripe mango fruit volatiles	In vitro	(*E*)-3-hexen-1-ol, (*E*)-2-octen-1-ol, phenylethyl alcohol, *p*-cymen-7-ol, (*Z*)-6-nonenal, 1-decene, indole, α-pinene, β-myrcene, α-phellandrene, 3-carene α-terpinene, *p*-cymene, d-limonene, terpinolene, α-copaene, β-elemene α-gurjunene, (*E*)-caryophyllene, humulene, δ-cadinene, (*E*)-limonene oxide, caryophyllene oxide	*Anopheles gambiae*	F	Terpinolene/humulene/humulene + (*E*)-caryophyllene + terpinolene	[153]
(E) Other						
Range of volatile chemicals	In vitro	3-Octanone, 2-nonanone, acetophenone, 4-propyl benzaldehyde, sulcatone, eugenol, 2,6,6-trimethyl-2-cyclohexene1,4-dione, dehydrolinalool, 4′-ethylacetophenone, γ-decanolactone, farnesene, gernayl acetate, geraniol, *R*-(+)-limonene, linalool oxide, hexyl hexanoate, hexyl-2-methylbutanoate, methyl benzoate, methyl 2-methyl benzoate, methyl salicylate, methyl cinnamate, *cis*-3-hexenyl acetate, nonanal, phenylacetaldehyde, *p*-anisaldehyde, 2-phenyl ethanol, 1-octen-3-ol, decanoic acid	*Aedes albopictus*	M	Attractive: decanoic acid Repellent: 2-nonanone, acetophenone, sulcatone, 2,6,6trimethyl-2-cyclohexanene-1,4-dione, 3,7-dimethyl-oct-6-en-1-yn-3-ol, 4′-ethylacetophenone, 4-propyl-benzaldehyde	[190]
*Metarhizium robertsii* colonised cadavers	In vitro	Longifolene	*Aedes albopictus* *Anopheles sinensis* *Culex pipiens*	Mixed	Longifolene	[175]

Opportunistic, generalist or zoophilic mosquito species may respond more strongly to non-human host odours. For example, *Ae. albopictus* is highly responsive to rat-derived VOCs, while *Culex* mosquitoes, primary vectors of West Nile virus and Japanese encephalitis, use cues such as nonanal and CO_2_ to identify avian and human hosts [63–70]. These insights enable the development of species-specific lure blends that reflect natural host preferences.

### Infected host odours

Because mosquito blood-feeding underpins pathogen transmission, the impact of pathogens on mosquito host-seeking behaviour is a crucial consideration when designing lures that target mosquitoes with vectoral capacity. Pathogens such as protozoans, nematodes and viruses have evolved to manipulate host odour profiles, altering the VOCs that guide mosquito attraction [71–73]. These pathogen-induced changes not only enrich our understanding of the mechanisms of these vectoral pathogens but also highlight specific semiochemicals that can be exploited to develop more effective lures [63].

For example, individuals infected with *Plasmodium falciparum*, the parasite responsible for malaria, emit altered VOC profiles that increase their attractiveness to mosquitoes [71, 72, 74, 75]. This shift includes increases in the release of aldehyde (e.g. octanal, nonanal, decanal and heptenal) and terpene (e.g. α-pinene, β-pinene: (±)-limonene) volatiles from red blood cells [71, 75–78]. Incorporating these compounds into synthetic blends has been shown to significantly increase trap efficacy. Notably, the addition of key aldehydes such as heptenal, octanal and nonanal to ‘healthy’ human odour profiles increases their attractiveness to *An. gambiae* [75]. Similarly, blends of aldehydes and terpenes associated with malaria parasite metabolism can double the capture rates of female *Anopheles* when added to a human odour base [78]. These findings demonstrate how pathogen-modified semiochemicals can be directly translated into improved attractant formulations for surveillance and control.

Comparable patterns are observed in other vector–pathogen systems, further supporting the utility of semiochemical-based approaches. For instance, individuals infected with *Mycobacterium ulcerans*, linked to *Buruli ulcers* and potentially transmitted by *Aedes* mosquitoes, emit distinct VOC profiles that increase mosquito attraction [79]. Identifying and replicating such pathogen-specific odour signatures could inform the design of targeted lures for diverse mosquito species [63, 73, 80]. More broadly, investigating co-evolved semiochemical interactions across vector–host–pathogen systems provide a framework for discovering novel attractant blends that are highly specific and effective [77, 81, 82]. Similar work to see whether other mosquito-borne diseases alter the volatile profiles of their hosts may offer similar formulations of blends more attractive to mosquitoes than infected hosts. Investigating whether co-evolution of semiochemical signatures exists within other vector–pathogen–host complexes could help develop attractant blends that enhance mosquito lures for specific disease systems and further elucidate the pathology that makes vector-borne diseases so widespread.

In conclusion, research into host-seeking odours highlights the central role of complex semiochemical blends in mediating mosquito host preference and attraction. Harnessing these cues provides a strong foundation for the development of more effective attractant lure for blood-seeking mosquitoes [83, 84]. However, formulating such lures remains inherently challenging. Highly complex and dynamic odour blends mediate host-seeking behaviour, often comprising dozens to hundreds of VOCs, where both composition and relative proportions are critical [51, 54, 63, 85]. Pathogen-induced modifications to host VOC profiles further emphasise how subtle shifts in odour composition and proportion can significantly alter mosquito behaviour [63, 73, 75, 77]. These findings reinforce the potential of exploiting host-associated semiochemicals to develop more effective attractant lures that mimic highly attractive individuals [49, 54, 84].

Advances in behavioural, analytical and electrophysiological screening have improved the identification of key behaviourally and physiologically active compounds within host odours, enabling more targeted blend design [46]. Approaches that move beyond single-source odours, such as combining individually screened, host-associated odours, have shown promise in overcoming some of these challenges [86, 87]. Nonetheless, successfully translating these findings into field-effective lures requires a deeper understanding of how mosquitoes integrate multi-component signals under natural conditions. This is especially important in environments where synthetic blends most compete directly with blood-feeding hosts, such as urban settings.

Overall, progress in leveraging host-seeking odours will depend on resolving the complexities of blend composition and perception, with particular attention to achieving combinations that are both biologically relevant and competitive in real-world contexts.

### Mating

Mosquito mating typically occurs shortly after adult emergence, often within swarming groups where males use visual and auditory cues to locate females. Females generally mate only once, becoming refractory to further mating, while males may copulate multiple times. Successful mating depends on the physiological maturity of both sexes, including male sperm development and female sexual receptivity [88]. Mosquito mating is primarily driven by behavioural and acoustic cues, with olfaction playing a more limited, short-range role in the process [89, 90]. While long-range mate location is typically achieved through females flying to male swarms located around visual landmarks, chemical signals contribute at closer distances.

Host-derived odours, such as CO_2_ and skin volatiles, can influence mating by shaping encounter rates between sexes. While these cues are well established in activating females over long distances during host-seeking, they have also been shown to attract males, particularly *Aedes* species, over long distances [91]. This aggregation of both sexes around hosts increases local mosquito densities, thereby enhancing opportunities for mating interactions [89, 90, 92, 93]. Males likely exploit these cues to locate blood-feeding females, with host odours reported to trigger behaviours such as lekking in *Ae. aegypti* [93, 94]. However, the expression of male attraction appears context-dependent. In the study by Amos et. al [91], male attraction to host cues was absent in confined laboratory assays but emerged under semi-field conditions, underscoring the importance of spatial scale and environmental realism in revealing natural mosquito behavioural responses. These findings highlight the potential for host-based odours to simultaneously target both sexes in trapping strategies.

Short-range olfactory cues involved in mosquito mating, including cuticular hydrocarbons and putative pheromones, contribute primarily to species recognition and mate assessment once individuals are already in close proximity. Although several contact sex pheromones such as (*Z*)-9-tricosene have been identified, their low volatility limits their effectiveness as long-range attractants and therefore constrains their direct application in lure development [95].

Some volatile compounds associated with mating behaviour have shown greater promise. In *Ae. aegypti*, swarming males release aggregation-related semiochemicals, including ketoisophorone and its analogue, which can induce female aggregation and enhance trap catches under laboratory and semi-field conditions [94, 96]. In these studies, males failed to respond to dead stationary ones, suggesting additional modalities are involved in triggering mating behaviour for males [96]. Similarly, in *Anopheles* species, swarming males emit volatile blends that attract both sexes and prolong swarm formation, with semi-field studies demonstrating increased mating success when these natural odour cocktails are present [97]. These findings suggest that olfactory cues play a greater role in *Anopheles* mating, while *Aedes* mosquitoes rely more on acoustic signals. Such species-specific differences influence reproductive isolation and could be exploited for species-targeted mosquito control using aggregation pheromones.

However, the utility of mating-related semiochemicals remains limited by their primary short-range activity and by the multimodal nature of mosquito mating behaviour. In contrast to host-seeking, where olfaction plays a dominant role, mating relies heavily on acoustic cues, with olfactory signals acting as supplementary rather than primary drivers of attraction [88].

Consequently, while mating-associated semiochemicals are unlikely to replace attractants in long-range lures, they may provide value in synergistic components in integrated strategies. In particular, they offer potential for enhancing trap efficiency at close range, improving sterile insect technique (SIT) programmes through increased mate encounter rates, or disrupting natural mating systems.

## Oviposition-associated odours

Oviposition represents a key behavioural stage that can be exploited for mosquito control, as gravid females actively seek suitable sites that maximise offspring survival. These decisions are mediated by the integration of visual, tactile and olfactory cues associated with aquatic habitats, including indicators of nutrient availability, competition and predation risk [21, 98]. These preferences vary among species according to egg morphology and laying behaviour, predation risk, and larval feeding ecology [98, 99]. After blood-feeding and during egg maturation, females undergo pronounced physiological and chemosensory changes that alter responsiveness and behaviour, enabling selective targeting by oviposition-based interventions [100].

### Conspecific odours as ovipositional cues

Gravid females are often attracted to established larval sites of their biotype by volatiles from conspecific eggs and larvae [101, 102]. These signals range from true pheromones (endogenously produced and species-specific) to environmentally derived semiochemicals. True pheromones, such as the *Culex* mosquito oviposition pheromone (MOP; erythro-6-acetoxy-5-hexadecanolide), are potent, highly specific stimulants for various *Culex* species, identified from apical droplets of *C. pipiens* eggs [51, 103, 104]. Similar to mating pheromones, the low volatility of MOP restricts its function to short-range attraction. In *Ae. aegypti*, conspecific-derived fatty acids such as dodecanoic acid, (*Z*)-9-hexanoic acid and hexanoic acid act as oviposition stimulants, although their ability to mediate long-range attraction remains unclear [105, 106]. This distinction between attraction and stimulation is critical; many compounds identified as ‘attractants’ may primarily function at close range, limiting their stand-alone utility in lure design.

The stage and density of conspecific larvae, as well as water from breeding sites, strongly mediate the attraction of gravid female mosquitoes [101, 102, 107]. In container breeders such as *Ae. aegypti*, volatiles from later instars can signal habitat persistence and attract oviposition at moderate densities, whereas very high larval densities deter females [107, 108]. In some *Anopheles* species, higher densities of early instars are attractive, but increasing densities of later instars become olfactory deterrents, reflecting trade-offs between habitat persistence and competition or cannibalism [109]. Identification of electrophysiologically active components of conspecific odours have identified blends which have been proven to be highly potent lures in laboratory-based assessments [107].

### Larval habitat microbes as ovipositional cues

Microbial semiochemicals represent a promising source of long-range oviposition attractants. Cedrol, a sesquiterpene alcohol detected in soil and water in natural *Anopheles* breeding sites, was the first semiochemical identified as an oviposition attractant for *An. gambiae* [110]. In field trials, traps baited with cedrol-enhanced lake water captured approximately three times more *Anopheles* and *Culex* females of various physiological stages than lake-water controls, with a disproportionate increase in gravid individuals [110]. Laboratory, semi-field and field studies confirmed that cedrol functions both as a long-range attractant and as an oviposition stimulant, boosting overall capture rates and increasing the proportion of gravid females responding.

Cedrol is produced by *Fusarium* fungi associated with plant rhizomes, such as *Cyperus rotundus*, commonly found in breeding habitats [111–113]. Its abundance declines following soil sterilisation, supporting its microbial origin, and members of the *Fusarium fujikuroi* complex are known to exploit insect vectors for dispersal. This raises the possibility that cedrol functions as an adaptive signal to attract gravid mosquitoes to microbe-rich habitats [114].

Bacterial volatiles from larval water, soil and oak leaf (*Quercus alba*) infusions significantly increased *Ae. albopictus* oviposition, with larval breeding water volatiles eliciting both the strongest behavioural and electrophysiological responses [115]. In contrast, soil and oak infusions induced behavioural attraction without detectable electrophysiological activity, suggesting that the discrepancy may be due to the collected volatiles being too dilute for electrophysiological detection [90]. This highlights a key limitation in relying solely on electrophysiology for identifying behaviourally relevant semiochemicals.

Evidence suggests that certain odours associated with larvicidal or predatory environments may function as ovipositional attractants under specific ecological conditions [116]. Microbial communities linked to predation of *Ae. aegypti* larvae by *Toxorhynchites theobaldi* produce volatiles that stimulate oviposition in *Ae. aegypti* [117]. Similarly, volatiles from *Bacillus thuringiensis israelensis* (Bti) and its filtrate show variable, context-dependent effects on oviposition in *Culex* and *Aedes* species [118–121]. These findings challenge the assumption that oviposition cues reliably indicate optimal habitats and suggest potential for ‘attract-and-kill’ strategies, although inconsistent responses currently limit their practical application.

### Plants as ovipositional cues

Plant-associated semiochemicals, often mediated by microbial decomposition, contribute another major class of olfactory oviposition cues. Vegetation linked to larval habitats provides both shelter and nutritional resources and emits volatiles that can guide oviposition site selection. For example, gravid *An. arabiensis* and *An. coluzzii* preferentially orient towards plant species associated with higher larval densities, including *Echinochloa* spp., *Typha latifolia* (Typhaceae) and *Cyperus papyrus* (Cyperaceae) [122], while habitat-associated grasses attract *An. albimanus* [123].

Detritus infusions, particularly those derived from hay, generate complex volatile profiles during decomposition that are highly attractive to *Aedes* and *Culex* species but often repel *Anopheles*, reflecting strong species-specific responses [23, 124–127]. For instance, *Cynodon dactylon* (Bermuda grass) stimulates oviposition in *Culex* and *Aedes* but deters *An. gambiae* females [125, 127, 128]. Sterilisation reduced the attractiveness of these infusions, confirming that microbial metabolism is a key driver of semiochemical production [96, 125, 129, 130].

The translation of plant-derived cues into practical lures has already demonstrated success. Volatile compounds such as nonanal, decanal and 3-methylphenol, derived from *Megathyrsus maximus* infusions (guinea grass), have been incorporated into the commercial attractant AtrAedes (Ecovec Ltda., Belo Horizonte, Brazil), which selectively targets *Aedes* mosquitoes [131–134]. More broadly, recent work has shown the value of integrating chemical ecology with thorough electrophysiology to rationally design synthetic blends. Studies on domesticated grasses (e.g. rice, maize and sugarcane), which are increasingly co-opted as mosquito breeding habitats, have identified electrophysiologically active volatiles and enabled the formulation of monterpene-rich synthetic blends [tab. 1C] [135–139]. Subtractive and combinatorial approaches revealed key components driving attraction, with chimeric blends outperforming single-sourced formulations in field trials by attracting both female and male *Anopheles* and *Culex* species [135–138]. These findings reinforce the importance of blend composition and synergistic interactions over individual compounds in achieving effective lures.

Additional semiochemical sources continue to expand the range of candidate attractants. Tea-derived infusions, particularly dilute, aged infusions of post-fermented Liu-pao tea, have recently been shown to act as potent oviposition attractants for *Aedes* mosquitoes [140]. Their activity persists following autoclaving, indicating that volatile phytochemicals produced during the manufacturing fermentation process, rather than microbial activity during infusion, are responsible. Cedrol, also present in these infusions, contributed to attraction; however, whole infusions consistently outperform individual compounds, further underscoring the importance of complex blends [140]. Similarly, geosmin, a cyanobacterial-derived sesquiterpenoid, has been shown to attract gravid *Ae. aegypti* and increase oviposition in both laboratory and field settings, with low-cost sources such as beetroot peel providing practical delivery mechanisms [141–143].

Despite their ecological relevance in aquatic habitats, aquatic photosynthetic organisms have received little attention as mosquito attractants or oviposition cues, as research on them has largely concentrated on the larvicidal potential of filamentous algae [144, 145]. Algae are present in most water sources suitable for mosquito larvae and serve as part of the larval diet, and so may be a source of odours attractive to both adults and juveniles [99, 146]. Algae such as *Spirogyra majuscala*, a filamentous alga found in mosquito breeding sites, have been shown to increase oviposition of *Anopheles pseudopunctipennis*; however, underlying chemical cues are unidentified [147].

Conversely, not all identified oviposition cues are suitable for lure development. Urea, for instance, increases oviposition in *C. tritaeniorhynchus*, but likely acts as a short-range gustatory stimulus rather than a volatile attractant, limiting its usefulness in spatial lures [148]. Urea is a component of many nitrogenous fertilisers, and so the implication of it as an ovipositional attractant, coupled with the widespread use of nitrogenous fertilisers, means that fertilised crops may become more attractive for gravid female mosquitoes.

Reverse chemical ecology approaches have identified individual ligands such as acetaldehyde that activate *C. quinquefasciatus* olfactory receptors and attract females across a range of concentrations [149]. While informative for understanding olfactory pathways, such single-compound approaches may fail to replicate the complexity required for robust field efficacy.

Collectively, these studies demonstrate that oviposition behaviour is governed by a complex interplay of conspecific, microbial, plant-derived and abiotic chemical cues that vary markedly across species and ecological contexts. While many identified oviposition attractants show strong species specificity and are tightly linked to larval habitat quality, emerging evidence indicates that some cues may influence a broader range of life stages or behaviours, expanding their potential utility beyond gravid females alone. A deeper understanding of how these oviposition cues are integrated within mosquito olfactory pathways will be essential for rational lure design and for maximising the effectiveness of oviposition-based interventions within integrated vector management strategies. Overall, evidence supports the potential for highly specific lures, but key gaps remain: the attraction range of many cues, consistency across habitat types and responses in virgin or non-gravid females, males and across genera.

## Foraging-associated odours

Adult male mosquitoes rely entirely on sugar-rich resources for energy and nutrition, while females supplement blood meals with plant-derived sugars, making sugar-foraging behaviour a promising but underexploited target for semiochemical-based lures [24]. They forage for plant sugars from nectars, fruits and honeydews, with sugar-source selection influencing mosquito lifespan, fecundity and pollination behaviours [100, 150–154].

Mosquitoes locate these resources using a combination of visual and olfactory cues, with plant-derived phytochemicals playing a central role, particularly for nocturnal species [100]. However, attraction is not determined solely by sugar content. Although mosquitoes preferentially feed in sugar-rich habitats, they do not consistently favour plants with higher sugar content [100, 155]. Instead, attraction appears to be driven by variation in phytochemical composition, including terpenoids, fatty acids, nitrogenous compounds and benzenoids, as well as microbial modification of nectar, which can alter both volatile profiles and nutritional quality [24, 151, 156, 157].

Current approaches to exploiting sugar-feeding behaviour, such as attractive toxic sugar baits (ATSB), not only demonstrate the potential of plant-derived lures but also highlight significant limitations. The effectiveness of ATSBs is often inconsistent owing to seasonal variation in plant resources and degradation of bait through microbial fermentation [158, 159]. These constraints underscore the need for chemically defined lures that replicate key attractive cues while maintaining stability under field conditions.

A growing body of research has sought to identify and synthesise such cues from floral odours. In *C. pipiens*, blends derived from host plants such as *Asclepias syriaca* (common milkweed) and Silene otitis (Spanish catchfly) have demonstrated strong attraction in both sexes, with key compounds including benzaldehyde, phenylacetaldehyde and (*E*)-2-nonenal [160–164]. Electrophysiological and behavioural studies indicate that simplified blends of a limited number of compounds can retain or even enhance attractiveness relative to full plant odours, although prior olfactory experience can modulate responses, suggesting a role of learning in sugar-foraging behaviour [163, 164]. Similar findings in *Abelia chinensis* (Chinese abelia) and other floral systems confirm that optimised multi-component blends often outperform single compounds, reinforcing the importance of synergy in lure design (Table 1D), [165, 166].

Comparable strategies have been applied to *Anopheles* mosquitoes, particularly in malaria-endemic regions [155, 167, 168]. The invasive plant *Parthenium hysterophorus* produces volatiles that attract both male and female *An. gambiae*, with synthetic blends derived from electrophysiologically active compounds performing as well as human odour cues and commercial lures in field trials (Table 1D), [155, 167, 169]. Different methodological approaches have been employed to identify behaviourally relevant compounds from complex floral headspaces. Nyasembe et al. [155] used gas chromatography–electroantennographic detection (GC–EAD) to identify volatiles eliciting antennal responses in females, whereas Nikbakhtzadeh et al. [167, 168] applied subtractive behavioural assays to determine which components are essential for attraction (Table 1D). While both approaches have yielded viable candidate blends, their relative effectiveness is difficult to compare owing to differences in experimental design and the behavioural metrics used to quantify attraction. This methodological inconsistency highlights a broader limitation in semiochemical research, where electrophysiological activity does not always translate directly to behavioural relevance. Given the widespread distribution of the source plant, further validation of these blends across multiple mosquito species, ecological contexts and against established lure formulations is needed to assess their robustness and practical applicability.

Individual phytochemicals can also exhibit strong behavioural effects, though their responses are often context-dependent. Compounds such as anisaldehyde, eugenol, linalool, geraniol, citronella and citral elicit attraction in *Aedes* females but may also interfere with other behaviours; for instance, anisaldehyde can inhibit host-seeking when combined with host odours [170]. Linalool and its derivatives illustrate both the potential and complexity of such cues. Linalool is a key component of a highly attractive three-component lure for both *Ae. aegypti* and *Ae. albopictus*, though each species exhibits distinct concentration preferences (Table 1D) [171]. This suggests that species specificity can be achieved through dose optimisation of shared blend structures [171].

Its derivative, linalool oxide, is similarly effective, acting as a potent attractant across multiple mosquito taxa [155, 163]. Field trials show that linalool oxide-baited traps captured *Aedes* at levels comparable to commercial host lures and performed even better when combined with CO_2_ [172]. Responses to linalool are also stereoisomer-specific in *An. gambiae* [173], indicating that molecular configuration, alongside concentration and blend composition, is critical for optimising lure efficacy.

Despite these advances, a substantial proportion of mosquito–plant interactions remains poorly characterised. Mosquitoes exploit a wide diversity of sugar sources, yet many of the key volatiles mediating these interactions are unidentified. Techniques such as DNA barcoding have begun to reveal the range of plant species utilised by wild *Aedes* populations, offering new directions for identifying candidate semiochemicals [174]. However, translating these ecological insights into effective lures remains challenging, as candidate blends must compete with abundant natural sugar sources and maintain efficacy under variable environmental conditions.

Overall, while sugar-derived semiochemicals offer considerable promise, particularly owing to their ability to attract both sexes across multiple species, their application in lure development is constrained by variability in plant chemistry, microbial influences and context-dependent behavioural responses. Progress will depend on identifying robust, ecologically relevant blends that retain attractiveness across environments, as well as improving formulation strategies to ensure stability and consistent release in field conditions.

## Natural enemy-associated odours

Non-plant biological systems are increasingly recognised as a source of behaviourally active volatiles exploited by mosquitoes. These cues arise from fungi, entomopathogens and microbial communities not directly linked to plant substrates or blood hosts, expanding the ecological breadth of semiochemicals relevant to mosquito behaviour.

Entomopathogenic fungi represent a notable emerging source of attractive volatiles. *Metarhizium robertsii*-colonised insect cadavers release longifolene, a sesquiterpene that attracts both male and female *Aedes albopictus* [175]. Building on this, Tang et al. [175] engineered a *Metarhizium* strain to constitutively produce longifolene, creating a single organism ‘lure-and-kill’ system capable of drawing in mosquitoes independently of human and plant odours. Although longifolene-mediated attraction competes with highly attractive floral volatiles such as those from *Ruellia brittoniana*, field and laboratory assays demonstrated that the engineered fungus achieved > 90% mosquito mortality, confirming the potential of fungal volatiles for integrated vector management. Importantly, these cues represent a departure from plant-derived signals: Rather than indicating resource-rich larval habitats, they function as broad-spectrum attractants exploitable for surveillance or population suppression.

Collectively, these studies demonstrate that mosquito attraction extends beyond cues tied to nutritional and reproductive benefit. Fungi, entomopathogenic bacteria and predator-associated microbial assemblages all contribute volatile cues that mosquitoes detect and exploit. By harnessing these non-plant biological signals, vector control strategies can diversify beyond traditional plant-based lures, enabling biocontrol-compatible attract-and-kill systems that operate independently of nutritional or reproductive substrates.

## Perspectives

Mosquito attractants represent a critical component of modern vector control, underpinning strategies ranging from surveillance and baited insecticide systems to SIT programmes. Because mosquito behaviour is highly context-dependent, cues associated with blood-feeding, mating, oviposition and sugar foraging offer opportunities for selectively targeting specific species, sexes and physiological states without relying on broad-spectrum population suppression. However, despite the identification of numerous candidate blends, their relative efficacy across taxa and ecological contexts remains insufficiently resolved, and few have been rigorously benchmarked against natural odour sources.

Progress in semiochemical discovery is strongly shaped by methodological approaches [161, 176]. Solvent extractions, headspace collection and electrophysiological techniques such as GC–EAD and single sensillum recordings each capture different aspects of the chemical landscape, yet none alone reliably predict behavioural relevance. The frequent mismatch between physiological detection and behavioural attraction highlights a central limitation: Mosquito responses are typically driven by complex, content-dependent blends rather than individual compounds. This issue extends to emerging approaches such as reverse chemical ecology, which provide valuable insight into receptor–ligand interactions but are inherently limited in their ability to identify multi-component blends that underpin robust attraction.

The importance of blend composition introduces additional challenges for lure development. Attractive odour profiles are shaped not only by compound identity but also by their ratios, concentrations, stereochemistry and temporal release dynamics, all of which are further modified by environmental factors such as temperature, humidity and ultraviolet (UV) exposure. Achieving stable and ecologically realistic release profiles in field settings remains a major challenge, given the susceptibility of semiochemicals to heat, humidity and UV-driven degradation. Replicating these conditions in field-deployable formulations remains a major obstacle, with compound degradation and inconsistent release rates often reducing lure performance. As a key result, the translation of laboratory-optimised blends into stable, competitive field lures remains a key bottleneck.

Emerging evidence further suggests that mosquito attraction is not static but can be modulated by factors such as prior experience and pathogen infection. Pathogen-induced changes in host odour profiles and mosquito sensory sensitivity present opportunities for targeted surveillance of infected vectors, although these approaches remain largely conceptual and require substantial field validation [177]. Pathogen-induced olfactory modulation suggests that attractants may need to be tailored not only to species but also to infection status, opening new avenues for surveillance tools capable of preferentially capturing infected, transmission-competent mosquitoes.

Across behavioural contexts, several key research gaps constrain progress. These include an incomplete understanding of the distinction between long-distance attractants and short-range stimulants, limited characterisation of synergistic interactions within complex blends and a continued reliance on laboratory-based assays that may not reflect field conditions. Many ecologically relevant semiochemical sources, including aquatic microbial communities and plant-associated systems, remain unexplored. Non-target impacts, production costs and the feasibility of scalable, controlled release technologies must also be assessed before widespread adoption. Regulatory frameworks for deploying semiochemical-based interventions, particularly genetically engineered lure systems such as longifolene-producing *Metarhizium* [175], will also require careful consideration.

Ultimately, advancing mosquito lure development will require a shift from single-compound discovery towards integrative, ecology-driven approaches that prioritise blend optimisation, controlled release technologies and robust field validation. Interdisciplinary collaboration, spanning chemical ecology, sensory biology, formulation chemistry and operational vector control, will be essential to bridge the gap between laboratory discovery and practical application. By addressing these challenges, semiochemical-based lures have the potential to become highly selective, scalable and sustainable tools within integrated vector management, capable of reducing mosquito populations and mitigating disease transmission in diverse ecological settings.

## Conclusions

Semiochemical-based mosquito control highlights the vast diversity of attractant types that can be exploited across the mosquito life cycle. These include host-derived odours for blood-feeding females, pathogen-altered host cues that enhance detection of infectious individuals, oviposition-site volatiles from conspecifics, microbes and plants, sugar-associated floral cues that attract both sexes, mating-related signals, and even non-traditional attractants from fungi or predator-associated systems. These sources offer a compelling and increasingly sophisticated pathway for enhancing mosquito surveillance and control by exploiting the chemical ecology underlying key behaviours, including host-seeking, mating, oviposition and sugar foraging. While substantial progress has been made in identifying behaviourally active compounds and developing synthetic blends, their practical implementation remains constrained by our understanding of the complexity of natural odour systems, variation across environments and species, and challenges in formulation and delivery under field conditions. The evidence consistently highlights that mosquito attraction is governed by dynamic, multi-component blends rather than single compounds, which can be selectively targeted to capture specific species, sexes or physiological states. Addressing these challenges will require interdisciplinary collaboration and a stronger alignment between laboratory findings and real-world ecological contexts. Ultimately, by refining our understanding of how mosquitoes perceive and respond to semiochemical cues, it will be possible to develop more selective, sustainable and effective interventions that complement existing control methods and contribute meaningfully to reducing the global burden of mosquito-borne disease.

## Data Availability

No new data was created during this study.

## Abbreviations

VOCs: Volatile organic compounds
GC–MS: Gas chromatography–mass spectrometry
Bti: *Bacillus thuringiensis* *israelensis*
SIT: Sterile insect technique
ATSB: Attractive toxic sugar bait
EAG: Electroantennography
GC–EAD: Gas chromatography–electroantennographic detection
OBP: Odorant-binding protein
